# Molecular and geographic analyses of vampire bat-transmitted cattle rabies in central Brazil

**DOI:** 10.1186/1746-6148-4-44

**Published:** 2008-11-05

**Authors:** Yuki Kobayashi, Go Sato, Nobuyuki Mochizuki, Shinji Hirano, Takuya Itou, Adolorata AB Carvalho, Avelino Albas, Hamilton P Santos, Fumio H Ito, Takeo Sakai

**Affiliations:** 1 Nihon University Veterinary Research Center, 1866 Kameino, Fujisawa, Kanagawa 252-8510, Japan; 2 Faculty of Agriculture and Veterinary Science, Department of Preventive Veterinary Medicine, UNESP, Via de Acesso Prof. Paulo Donato Castellane, Jaboticabal, São Paulo 14884-900, Brazil; 3 São Paulo State Agency of Agribusiness (APTA) – São Paulo State Secretary of Agriculture and Supply, Rod. Raposo Tavares, Km 563, Presidente Prudente, SP, Brazil; 4 School of Veterinary Medicine, Maranhão State University, Campus I, Cidade Universitária Paulo VI, Tirirical, Caixa Postal, 09 São Luís, Maranhão, Brazil; 5 Department of Preventive Veterinary Medicine and Animal Health, Faculty of Veterinary Medicine and Zootechny, University of São Paulo, Av. Prof. Dr. Orlando Marques de Paiva, 87, Cidade Universtiátria, São Paulo 05508-000, Brazil

## Abstract

**Background:**

Vampire bats are important rabies virus vectors, causing critical problems in both the livestock industry and public health sector in Latin America. In order to assess the epidemiological characteristics of vampire bat-transmitted rabies, the authors conducted phylogenetic and geographical analyses using sequence data of a large number of cattle rabies isolates collected from a wide geographical area in Brazil.

**Methods:**

Partial nucleoprotein genes of rabies viruses isolated from 666 cattle and 18 vampire bats between 1987 and 2006 were sequenced and used for phylogenetic analysis. The genetic variants were plotted on topographical maps of Brazil.

**Results:**

In this study, 593 samples consisting of 24 genetic variants were analyzed. Regional localization of variants was observed, with the distribution of several variants found to be delimited by mountain ranges which served as geographic boundaries. The geographical distributions of vampire-bat and cattle isolates that were classified as the identical phylogenetic group were found to overlap with high certainty. Most of the samples analyzed in this study were isolated from adjacent areas linked by rivers.

**Conclusion:**

This study revealed the existence of several dozen regional variants associated with vampire bats in Brazil, with the distribution patterns of these variants found to be affected by mountain ranges and rivers. These results suggest that epidemiological characteristics of vampire bat-related rabies appear to be associated with the topographical and geographical characteristics of areas where cattle are maintained, and the factors affecting vampire bat ecology.

## Background

Rabies is a fatal infection of the central nervous system caused by being bitten by a rabid animal. The vampire bat, which has a distribution extending from Mexico to Argentina, is an important rabies vector in the region. Outbreaks of rabies in livestock transmitted by vampire bats were first observed between 1906 and 1908 in the State of Santa Catarina in Brazil, when approximately 4000 cattle and 1000 horses and mules died due to paralytic rabies [1]. To date, cattle losses attributed to vampire bat transmitted rabies have had a marked economic impact on the livestock industry in the areas [1-3]. In addition, outbreaks of human rabies transmitted by vampire bats in the Amazon regions of Brazil are an important public health consideration [4-6].

The vampire bat is a non-migratory colonial species that roosts in natural shelters or in shelters associated with human habitation [7]. Since vampire bats feed on mammalian blood, the distribution of vampire bat populations is affected by food availability and the distribution of livestock and cattle in particular [7,8]. Consequently, the occurrence of rabies within vampire bat populations is very closely reflected by the incidence of rabies in cattle [3,9,10].

Vampire bat-related rabies viruses have been genetically typed as being a species-specific variant [11]. To date, several rabies virus variants have been identified based on geographic distributions, which possibly reflects their association with bat ecology [12-14].

Attempts to control the transmission of vampire bat-transmitted rabies by reducing the vampire bat population using warfarin and vaccinating livestock against rabies are regularly conducted in Brazil [1,2]. Knowledge of the epidemiological characteristics of vampire bat-transmitted rabies is thus important for assessing the efficacy of these control measures against rabies transmission by bats. In order to assess the epidemiological characteristics of vampire bat-transmitted rabies, the authors conducted phylogenetic and geographical analyses using sequence data of a large number of cattle rabies isolates collected over a wide geographical area in Brazil.

## Methods

The 570 rabies virus isolates genetically analyzed in this study were collected from cattle in the city of Brasília in the Federal District (DF) (n = 2), and the States of Goiás (GO) (n = 320), Maranhão (MA) (n = 18), Minas Gerais (MG) (n = 3), Mato Grosso do Sul (MS) (n = 64), Mato Grosso (MT) (n = 72), Pará (PA) (n = 1), Paraíba (PB) (n = 5), Rio de Janeiro (RJ) (n = 46), São Paulo (SP) (n = 22), and Tocantins (TO) (n = 17) between 1987 and 2006 (Accession numbers: AB307066–AB307631, AB377125–AB377128). In addition, 96 rabies virus sequences, which were analyzed in previous studies [11,12,15], were obtained from isolates of rabid cattle in the DF (n = 1), and the States of GO (n = 32), MA (n = 1), MT (n = 26), MG (n = 4), PB (n = 14), PA (n = 1), Rondônia (RO) (n = 1), SP (n = 9), and TO (n = 7) (Accession numbers: AB083799, AB083803, AB083805, AB083809, AB083813, AB083814, AB083818, AB206423–AB206436, AB246194–AB246210, AB246213–AB246248, AB246250–AB246267, AB246268–AB246270, AB307065). Eighteen rabies virus sequences were obtained from vampire bats in the States of GO (n = 10), RJ (n = 3), and SP (n = 5) as described previously (Accession numbers: AB201803–AB201805 and AB297632–AB297646) [16,17]. The nucleotide sequences of BRdg10 and BRdg603 isolated from Brazilian dogs were obtained from GenBank (Accession numbers: AB083796 and AB263334) [18].

Viral RNA was extracted from the brains of cattle diagnosed as being rabies positive by both the direct fluorescence antibody test and the mouse inoculation test [19,20]. The mice were housed and handled with ethical principal under the committee of University of São Paulo.

RT-PCR and sequencing methods were as described previously [12]. A 203 nt region corresponding to the nucleoprotein gene located between nucleotide 109 and 311 of the PV strain was analyzed as this locus has been employed to demonstrate association with the phylogenetic divergence of clusters in previous phylogenetic studies [11,21].

Multiple alignment and phylogenetic analysis were performed using the Clustal × program [22]. Phylogenetic trees were generated with the p-distance model using the neighbor-joining method of Saitou and Nei [23] and the Mokola virus was used as an outgroup (Accession number: Y09762). The statistical significance of the constructed phylogenies was estimated by bootstrap analysis with 1000 pseudoreplicate datasets. Bootstrap values exceeding 70% were considered to indicate phylogenetic association [24]. The TREEVIEW program was used to obtain a graphical output [25] and nucleotide sequence identities were calculated using BioEdit software [26]. The geographic origins of the sequenced Brazilian cattle and vampire bat rabies isolates were plotted at the municipal level of the respective federal states using MapInfo Professional GIS software (ver. 8.0 software MapInfo Japan K.K., Tokyo, Japan).

## Results

**Figure 1:**
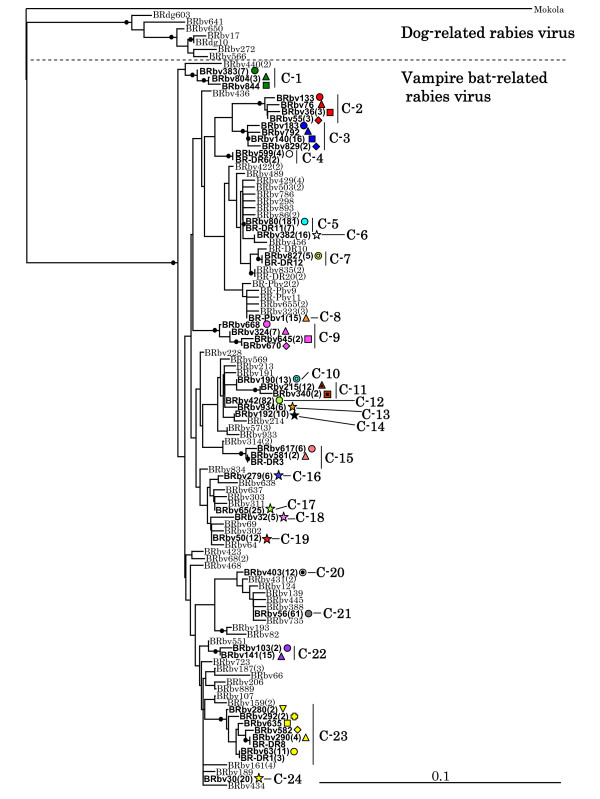
**Distance tree generated by the neighbor-joiningmethod**. Mokola virus was used as an outgroup. Bootstrap values were calculated with 1,000 iterations, and black circles indicate internal branches with bootstrap values of ≧70%. Letters of BRbv, BRdg, and BR-DR indicate samples from Brazilian cattle, dogs and vampire bats, respectively. The number of samples exhibiting 100% nucleotide identity is shown in parenthesis. The symbols corresponding to the phylogenetic clusters with a ≧70% bootstrap value on internal branches, and the clusters consisting of more than five samples, C-1 – C-24, are shown in this figure.

Brazilian rabies viruses could be divided into two phylogenetic groups, dog- and vampire bat-related rabies virus variants (Fig. 1). Almost all (99.2%) of cattle isolates analyzed in this study were identified as being the vampire bat-related rabies virus variant, which consisted of a large number of phylogenetic lineages, the other five cattle samples collected from the States of GO, MA, MT, and SP during 1995–2004 were characterized as being dog-related rabies virus variants.

**Figure 2:**
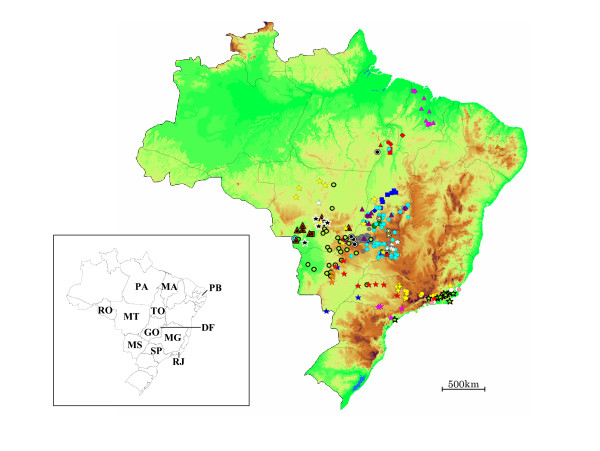
**Geographic distribution of cattle isolates classified as genetic variants in this study**. City and state abbreviations are as follows: DF, Brasília City in the Federal District; GO, Goiás State; MA, Maranhão State; MG, Minas Gerais State; MT, Mato Grosso State; MS, Mato Grosso do Sul State; RJ, Rio de Janeiro State; RO, Rondônia State; PA, Pará State; PB, Paraíba State; SP, São Paulo State; TO, Tocantins State. The symbols for the cattle isolates correspond to those used in figure 1. Samples for which the geographic origin and the genetic variant are identical are illustrated using the same symbol.

**Figure 3:**
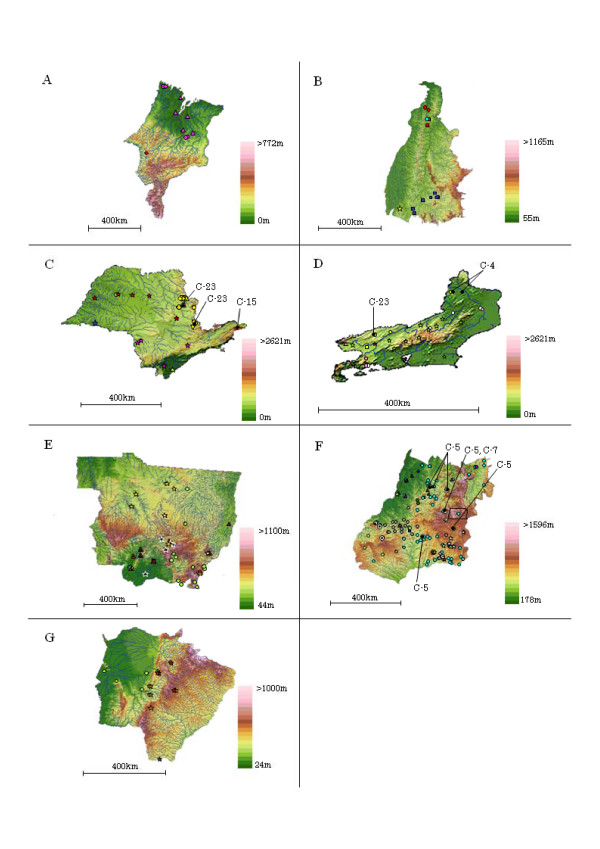
**Detailed geographic distribution of cattle and vampire bat isolates classified as genetic variants**. (A)Maranhão State, (B)Tocantins State, (C)São Paulo State, (D)Rio de Janeiro State, (E)Mato Grosso State, (F)Goiás State and Brasília City of the Federal District, and (G)Mato Grosso do Sul State. Closed circles indicate the geographic origins of vampire bat isolates classified as genetic variants. Other symbols correspond to those of the cattle isolates used in figure 1. Rivers appear as blue lines. Samples for which the geographic origin and the genetic variant are identical are illustrated using the same symbol. Brazilian maps were obtained from Brasil em Relevo – Embrapa Monitoramento por Satélite .

**Figure 4:**
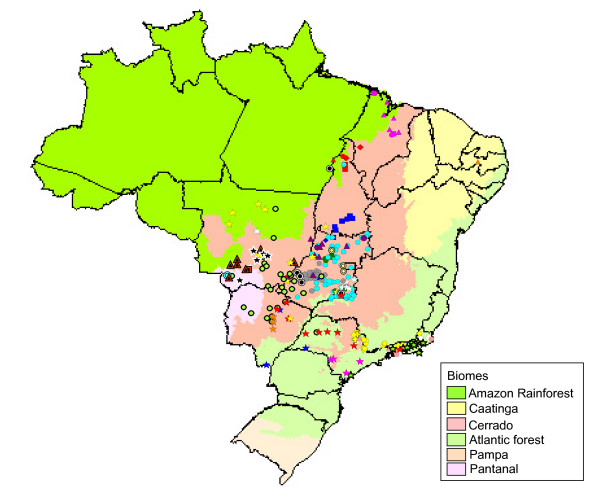
**Geographic distribution of cattle isolates classified as genetic variants in Brazilian biomes**. Symbols correspond to those used in figure 1. The Brazilian biome map was obtained from IBGE Mapas Interativos .

To assess geographical distribution pattern of the virus, vampire bat-related cattle isolates belonging to clusters with bootstrap values at the shared internal branches of lineages exceeding 70% (Fig. 1), and clusters consisting of more than five samples with 100% nucleotide similarity were plotted on topographical Brazilian maps. Consequently, 593 samples belonging to 24 clusters, C-1 – C-24, were mainly analyzed and plotted in maps (Fig. 2, 3, 4). The nucleotide sequence identities among 24 clusters ranged between 93.1 – 99.5%. C-1 – C-3, C-9, C-11, C-15, C-22 and C-23 consisted of several lineages with bootstrap values exceeding 70%. The nucleotide sequence identities within these clusters ranged between 97.5 – 99.5%, and the distributions of the samples belonging to these clusters were observed to overlap (Fig. 2).

Most of the samples analyzed in this study were found along rivers, and a large number of samples characterized as the same variant were observed to be distributed along the same river (Fig. 3). Although the distributions of several clusters overlapped, regional variations in the distributions of these clusters were observed in Brazil (Fig. 2). C-2, C-8 and C-9 were mainly distributed in the northern regions, C-1, C-3, C-5 – C-7, C-10 – C-14, C-20 – C-22 and C-24 in the central regions, and C-4, C-15 – C-19 and C-23 in the southern regions of Brazil. In other 86 cattle samples which were not categorized by the above-mentioned method, the distributions of the samples with 100% similarity were also observed in adjacent geographic areas (data not shown).

The distributions of several clusters were separated by geographic boundaries such as mountain ranges. For example, mountain ranges in the States of GO, MT and MS were observed to separate C-24 in the north and northwest, C-10 – C-12 and C-14 in the southwest areas, C-5 and C-20 – C-22 in the northeast areas, and C-13, C-16 and C-19 in the southeast (Fig. 2). In addition, the distributions of C-1, C-3, C-5 and C-22 were affected by the mountain ranges in the eastern areas of the State of GO (Fig. 3-F). C-21 localized in a region between two mountain ranges in the State of GO. C-4 and C-17 were mainly distributed in the State of RJ, and were surrounded by the mountain ranges in the States of MG, SP and RJ with altitudes of 800 m above (Fig. 3-D).

Elevations in distribution regions of clusters defined in this study were various heights. For example, C-5, C-7, C-11, C-12, C-14, C-19 and C-23 were dispersed in mountain regions at altitudes 1000 m above to its surrounding lowland regions at altitudes 100 m below (Fig. 3). C-2, C-4, C-9 and C-22 were distributed in lowland regions at altitudes less than 300 m, and C-6 was primarily distributed at an altitude of 700 m in mountainous area of the State of GO (Fig. 3-F).

The effect of the different Brazilian biomes on the distribution patterns of bat rabies isolates was also assessed (Fig. 4). C-2, C-9, C-11, C-12, C-14, C-19 and C-24 were found to be distributed across several regions with different biomes, while other clusters tended to be restricted to regions with a particular biome.

Using the above-mentioned method of categorization, the 10 vampire bat isolates in 18 categorized were genetically classified as falling into C-4, C-5, C-7, C-15 and C-23. The distributions of the vampire bat isolates belonging to C-5, C-7 and C-23 overlapped with cattle isolates of the same lineage (Fig. 3-C, D, F). Although vampire bat isolates belonging to C-4 were isolated in remote areas several tens-of kilometers from the geographic origins of cattle isolates belonging to the same lineage, several vampire bat and cattle isolates were distributed along the same river (Fig. 3-D). A vampire bat isolate belonging to C-15 was isolated in adjacent geographic areas where the cattle isolates were classified as the same variant (Fig. 3-C).

The number of cattle isolates of C-2, C-6, C-17 and C-20 in remote areas was small compared to the size of the groups from the principal distribution regions (Fig. 2). While the majority of the samples belonging to C-6 and C-20 were mainly distributed in the mountain regions of the State of GO, one sample belonging to C-6 originated from the central lowland region in the State of MT and one sample in C-20 from the southeastern region of the State of PA were also observed. While C-17 consisted mainly of samples from the State of RJ, one sample was derived from the southern area of the State of SP. Similarly, while C-2 primarily contained samples from the northern lowland areas of TO, one sample was derived from the southeastern mountainous region of MT.

## Discussion

The previous research reported the existence of nine regionally-defined rabies virus variants in Brazilian vampire bats [12]. The distribution patterns of these variants were observed to differ with respect to the geographic origins of the viruses and suggested that the epidemiological characteristics of the rabies virus variants were associated with the ecology of the bat populations.

Vampire bats usually select the shorter routes between the roost and their preferred prey, often traveling several kilometers one-way to find prey [7]. In addition, although vampire bat population in a colony remains relatively stable, they regularly visit neighboring roosts within activity area of ranges of 10 to 20 km^2 ^[7,9,27]. This tendency to visit neighboring colonies promotes indirect communication between almost all of the colonies within an area forming a loosely defined assemblage or network [28]. Given these aspect of bat ecology, the spread of rabies in this species is mainly due to contact between the infected individuals of one colony and the susceptible individuals of another [8,28]. Rabies outbreaks in vampire bats have been observed to move slowly at an average rate of 40 km per year [3].

Although the categorization of a genetic group employed in this study differed from that employed previously [12], a large number of regionally-differentiated rabies virus variants were identified, and were widely distributed in both previously-studied and new areas of Brazil. The distributions of the variants identified in this study were observed to differ between regions in Brazil and tended to be separated by mountain ranges, which corroborated the observations of Kobayashi *et al*. and Velasco-Villa *et al *[12,13]. In addition, since the distributions of the classified genetic variants from both vampire-bat and cattle isolates were found to overlap relatively closely, the epidemiological characteristics of cattle rabies are markedly similar to those of vampire bat rabies, with the genetic diversity between these variants attributed to aspects related to bat ecology and the topographical features of the habitat. Consequently, it appears that the rabies viruses might be circulated among bat populations inhabiting in contiguous areas, and although the variant distributions may change slowly over time, natural barriers such as mountain ranges may play an important role in delimiting rabies foci, resulting in regionally-differentiated rabies virus variants. A similar geographic orientation has been observed in rabies virus variants transmitted within populations of other non-migratory bat species, i.e. *Eptesicus fuscus *in North America [29].

The authors previously reported that genetic variants of vampire bat-related rabies in mountainous regions were correlated with altitude [12]. However, in this study, several variants were observed to disperse across of the potential altitude barriers defined previously, moving from high altitude mountainous regions to lowland. In addition, the distributions across several distinct biomes were also observed. Vampire bats utilize rivers to move between areas, and paths of outbreaks have been observed to follow tributaries and splits in the river due to the abundance of suitable roosts and caves along their banks [28]. Consequently, the presence of rivers and adequate shelter has been recognized as important considerations underlying the incidence and spread of vampire bat-transmitted cattle rabies. Most of the samples analyzed in this study were isolated from adjacent areas linked by rivers, which agreed with epizootic observations in cattle rabies. In addition, a large number of samples characterized as the same variant that was distributed along the same river. These findings suggest that the distribution patterns of variants may correlate with the behavior of vampire bats, and that the spread of the virus is associated with bat migration.

In this study, a small number of variants were isolated in areas that were relatively remote and removed from the principal areas. Similarly, distribution patterns of the virus have been also observed that appear to indicate the movement of either vampire bats or infected animals across several Brazilian regions [14]. 86 of the samples in this study could not be categorized as genetic variants, and surveillance of cattle rabies has not yet been performed in several Brazilian regions. Subsequent epidemiological studies are, therefore, necessary in order to assess the temporal and spatial characteristics of the bat rabies distribution and also to obtain additional detailed epidemiological information related to bat rabies.

Since the epidemiological characteristics of vampire bat-transmitted rabies in cattle may be associated with topographical and geographical features of the areas where cattle are maintained as well as aspects of bat ecology, it is important that these factors are considered in plans directed at mitigating the spread of bat-transmitted cattle rabies.

## Conclusion

The present study revealed that several dozen of regionally-defined rabies virus variants associated with vampire bats exist in Brazil, and that areas where rabies occurs were affected by mountain ranges and rivers. These findings suggest that epidemiological characteristics of vampire bat rabies may be influenced by the topographical and geographical features of the areas in which cattle are maintained as well as aspects related to bat ecology. Consequently, the authors propose that these factors should be considered in measures directed to control the bat-transmitted cattle rabies.

## Competing interests

The authors declare that they have no competing interests.

## Authors' contributions

YK, carried out the molecular genetic studies, and edited the manuscript. GS, NM and SH, conducted the RT-PCR and the sequencing reactions. TI, AABC, AA, HPS, FHI and TS, participated in the elaboration of the study design, management, coordination, and assisted drafting the manuscript. All of the authors have read and approved the final manuscript.
